# Long-Term Sequela of Intrathecal Gadolinium Extravasation: Symptoms Mimicking Post-concussive Syndrome

**DOI:** 10.7759/cureus.14084

**Published:** 2021-03-24

**Authors:** Aakaash Patel, Anna Zolyan, Ahmed Itrat

**Affiliations:** 1 Department of Neurology, Northeast Ohio Medical University, Rootstown, USA; 2 Neurology, University of California Irvine Medical Center, Orange, USA; 3 Neurology/Stroke, Cleveland Clinic Akron General, Akron, USA

**Keywords:** gadolinium, toxicity, neuroradiology, toxic encephalopathy, post-concussive syndrome, neurology, radiology

## Abstract

Gadolinium contrast administration, usually with magnetic resonance imaging, is an important diagnostic modality in the investigation of neurological pathologies. There is little evidence in the literature suggesting repeated exposure to intrathecal gadolinium results in symptoms mimicking post-concussive syndrome (PCS). We studied one patient who received intrathecal gadolinium to investigate a pain pump malfunction and presented with encephalopathic symptoms of confusion and aphasia with imaging consistent with intracranial gadolinium extravasation. The patient was followed up regularly with repeat imaging, reassessment of persistent symptoms, and specialist evaluations; however, symptoms remained refractory and resembled PCS. Our findings indicate a need to further investigate potential associations between intrathecal gadolinium exposure and a clinical presentation consistent with PCS, irrespective of histopathological changes.

## Introduction

Repeated exposure to intravenous gadolinium is thought to have a cumulative effect over time with brain deposits that have been histopathologically confirmed in patients with normal renal function. Acute extravasation of gadolinium contents mimicking acute subarachnoid hemorrhage has also been described elsewhere, especially after intrathecal administration, including the patient in our case report [1,2]. There is, however, no clear consensus yet if these acute “high-dose” or gradually accumulating deposits result in long-term neurological sequelae. Recent studies have demonstrated gadolinium toxicity in preclinical in-vitro studies by pathophysiological mechanisms involving calcium homeostasis [3]. Once within the extracellular space, gadolinium is proposed to have affinity for certain other similar metals with resulting chelation and deposition in the central nervous system [4]. Animal studies have demonstrated increased markers of cellular injury after exposure to higher concentrations of gadolinium [5]. However, causal relationship is difficult to establish between gadolinium administration and neurological sequela [3]. We had the unique opportunity to study one such patient with acute toxic encephalopathy over a period of two years and report subsequent clinical findings.

## Case presentation

**Figure 1:**
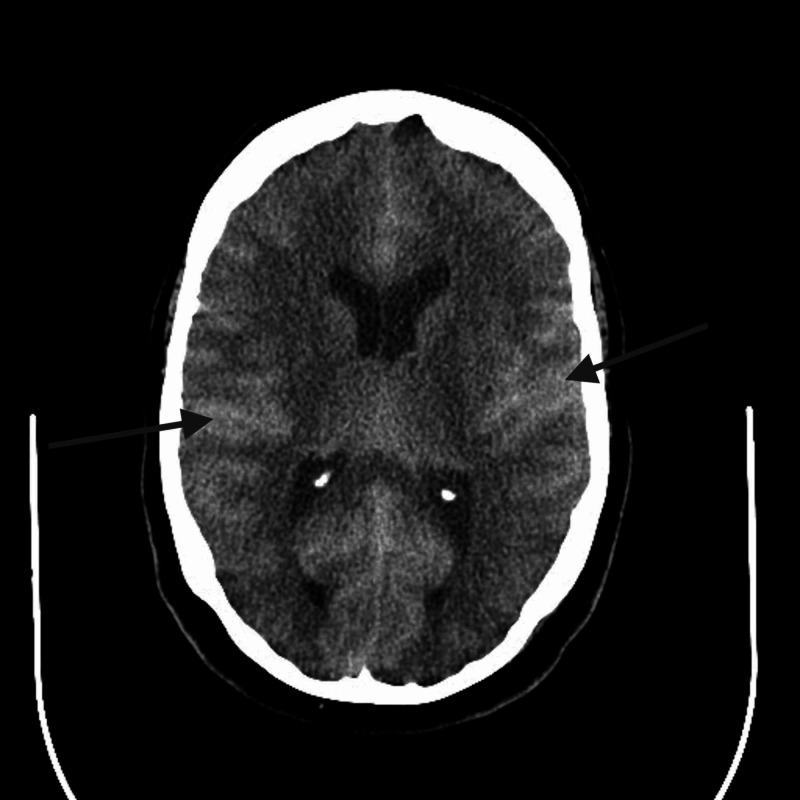
Non-contrast enhanced CT of the brain demonstrating subarachnoid hyperdensity mimicking appearance of a diffuse SAH (arrows point at hyperdensities). CT, computed tomography; SAH, subarachnoid hemorrhage

A 57-year-old male with normal renal function presented from an outside hospital due to acute confusion and aphasia following intrathecal gadolinium injection for a diagnostic myelogram to evaluate for a pain pump malfunction. A computed tomography (CT) scan of his brain revealed hyperdensity in basal cisterns which was initially thought to be a diffuse subarachnoid hemorrhage (SAH) (Figure 1). Cerebrospinal fluid (CSF) analysis was negative for xanthochromia, and demonstrated mild pleocytosis (11 white blood cells/mm^3^), normal proteins (42 mg/dL), and glucose (76 mg/dL). The combination of CSF findings, relatively mild symptoms with absence of angiographic findings, and recent intrathecal contrast administration was suspicious for diffuse gadolinium extravasation rather than SAH. This presentation was described in a separate paper [2]. Patient was discharged home after a relatively short stay and established follow-up care in outpatient neurology practice. His symptoms at discharge included mild aphasia, disorientation, and visual blurriness.

In his first follow-up about two months post-discharge, he reported making slow progress. He had multiple complaints which included persistent binocular diplopia, more pronounced on sudden head movement for which he was prescribed an alternating eye patch. The patient complained of constant tinnitus in both ears, head pressure sensation and circumferential headaches, disorientation and loss of focus with subtle head motion, tiredness, and fatigue. A repeat CT head at this time showed no abnormality and resolution of all contrast material. In addition, he complained of difficulty with multitasking and getting easily overwhelmed. Cognitive and occupational therapy also overwhelmed the patient when asked to engage in focus-directed tasks. Multiple electroencephalograms were negative for evidence of ictal activity during hospitalization and follow-up visits. A videonystagmogram did not reveal evidence of nystagmus. Patient performed well on the screening questionnaire for cognitive impairment and was therefore not referred for a full neuropsychiatric assessment evaluation.

**Figure 2:**
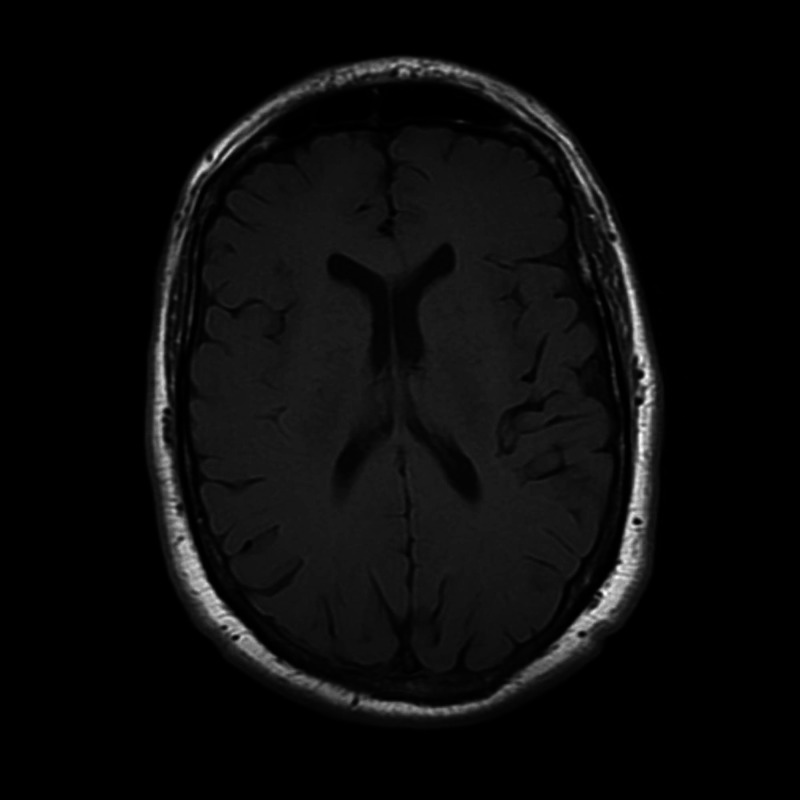
Follow-up non-contrast-enhanced MRI brain obtained one year following the initial event, with T1-weighted image showing absence of any deposits. MRI, magnetic resonance imaging

The patient continued follow-up for persistence of these non-specific symptoms which resembled post-concussive syndrome (PCS). Attempts to manage his symptoms with conventional PCS pharmacological treatment did not show much improvement. He also had intolerance to medication side effects. Therapies tried thus far included oral nortriptyline, duloxetine, trazodone, valproic acid, magnesium oxide, memantine, and methylphenidate for relief of multiple symptoms, which comprised headaches, motion sickness, anxiety, tinnitus, and sensitivity to multiple visual stimuli. A neuro-ophthalmology evaluation for diplopia was inconclusive. Occupational therapy referral for visual rehabilitation was also poorly tolerated. A follow-up MRI brain without contrast performed approximately one year after his initial injury did not demonstrate evidence of abnormal gadolinium deposits or other abnormal findings (Figure 2). At his most recent evaluation which was close to 24 months following his initial event, symptoms of headaches, tinnitus, and sensitivity to visual stimuli were all present.

## Discussion

The presented case represents a treatment challenge based on multiple subjective symptoms developing after gadolinium extravasation. While few case reports have described such cases [6,7], long-term sequelae or follow-up are not available for patients with gadolinium toxicity. In brief hospital studies, transient encephalopathy following contrast administration in patients with end-stage renal disease is described, although it does not appear there are lasting consequences in these studies [8]. Our patient’s symptoms curiously mimicked those of PCS, a disorder which has been difficult to define due to the subjective nature of symptoms, but characterized as a constellation of non-specific somatic symptoms including dizziness, nausea, vision and auditory disturbances, fatigue, and frustration last at least three months after a head injury [9]. The similarity of symptoms in our case to PCS suggests a potentially common pathophysiological mechanism or shared pathway, although lack of response to all forms of treatment is unusual. Pathophysiological mechanisms underlying gadolinium neuro-toxicity are not well described in literature, and animal studies have revealed varied clinical signs and features such as myoclonus, ataxia, tremor, with pathological findings of corpus callosum damage and hemorrhage after direct intra-ventricular injections [10]. In patients with renal failure, dentate nucleus and globus pallidus seem to be preferential deposition sites for gadolinium, as seen in cases of nephrogenic systemic fibrosis, although clinical significance of these deposits is unclear. An important disorder that can shed light on gadolinium deposition over time is multiple sclerosis, where frequent contrast-enhanced MRIs can result in excessive lifetime exposure to gadolinium. Once again, subcortical structures such as globus pallidus, dentate nuclei, and the thalamus were most frequently associated with gadolinium deposits without associated clinical features [11]. In our review of literature, no case reports describing visual pathway deposits with gadolinium exposure were found. Functional damage to the visual association cortex could be hypothesized as a possible mechanism. Our case is the only report that we could find describing long-term symptoms in a patient with this rare form of gadolinium extravasation, and we do not feel a single case establishes causality, but temporal association of patient’s new symptoms following this toxic exposure is intriguing.

## Conclusions

Gadolinium neurotoxicity is a rare potential complication of gadolinium-enhanced MRI use for brain imaging, as exhibited by direct cerebral injury following intrathecal administration. Although this solitary case does not demonstrate causality, further such descriptions may point toward a possible association, and further research is needed to determine if exposure to intrathecal gadolinium is associated with long-term neurological sequela, with or without abnormal deposits on brain imaging.
